# Dietary Exposure to Acrylamide in Spanish University Canteens by the Duplicate Diet Method

**DOI:** 10.3390/foods12234263

**Published:** 2023-11-25

**Authors:** Lucía González-Mulero, Cristina Delgado-Andrade, Francisco J. Morales, Marta Mesías

**Affiliations:** Institute of Food Science, Technology and Nutrition (ICTAN-CSIC), E-28040 Madrid, Spain; lucgon03@gmail.com (L.G.-M.); fjmorales@ictan.csic.es (F.J.M.); mmesias@ictan.csic.es (M.M.)

**Keywords:** acrylamide, dietary intake, university students, university canteen, dietary exposure, risk assessment

## Abstract

During the university period, many students adopt new dietary patterns, sometimes including the excessive consumption of highly processed foods, which can expose them to process contaminants such as acrylamide. This research aimed to evaluate the dietary exposure to acrylamide of Spanish university students in their campus canteens using the duplicate diet method, and to estimate the associated health risks based on their food consumption habits. Apart from potato-based foods, the analysed food/meals contained non-detectable or low levels of acrylamide (<63 µg/kg). Two exposure scenarios were considered, depending on whether students selected salad (lowest exposure) or processed potatoes (highest exposure) as side dishes. The frequent consumption of processed potatoes could increase acrylamide intake from 6.5 to 26.4 µg/day. Due to their lower body weight, women had a higher risk of acrylamide exposure in both scenarios. The margin of exposure (MOE) values for neoplastic effects indicated high levels of health concern, even only considering the main meal of the day. Furthermore, the risk of acrylamide exposure could increase by more than four times depending on the side dish selected by the students. This research highlights the need to promote healthy dietary habits among young people, encouraging the selection of safer food options in terms of food processing contaminants.

## 1. Introduction

In recent years, there have been significant changes in economic and sociocultural factors that have greatly influenced dietary habits in developed countries. Younger generations, including university students, are increasingly adopting an urban lifestyle characterised by a fast-paced lifestyle and a lack of time to learn traditional and healthier forms of cooking. As a result, this population group has become more dependent on catering and canteens companies, restaurants, and other food supply services [1]. During the university period, it is common for students to leave home and develop new daily habits that modify their dietary patterns. This can often result in a move away from a balanced diet due to a lack of experience in making healthy decisions or a low perception of the importance of proper nutrition. In that sense, students who eat in university canteens where buffet-style food is offered may not always choose the most recommended options. These decisions can have a significant impact on their health not only during their time at university but also in their future lives [2].

Political and social agents are making efforts to promote healthy eating habits by introducing changes in the knowledge, attitudes, and preferences of consumers. Furthermore, health authorities have increased control over the menus offered by public services to protect the health and well-being of the population. This includes implementing healthy menus and improving the nutritional profile by limiting the availability of sugary and fatty foods and increasing the provision of vegetables and fruits [3,4]. However, sometimes, the menus provided at certain public food establishments and institutions lack variety and include in most cases processed foods. In such instances, the choices available to the consumer are limited.

The consumption of processed foods can lead to exposure to unhealthy compounds known as chemical process contaminants, such as acrylamide. Acrylamide is a chemical compound that can naturally form during the Maillard reaction, which occurs when free amino acids, such as asparagine, react with reducing sugars, such as glucose and fructose [5,6]. In 1994, the International Agency for Research on Cancer (IARC) classified acrylamide as a probable human carcinogen (Group 2A) [7]. This was later supported by the European Food Safety Authority (EFSA) in 2015, when they indicated that dietary exposure to acrylamide may be a concern for neoplastic effects based on evidence from animal studies [8]. Given the potential risks associated with dietary exposure to acrylamide, it is important to estimate the levels of exposure in order to assess the associated risks. In this sense, the margin of exposure (MOE) approach provides an indication of the level of health concern about the acrylamide presence in foods without quantifying the risk. The use of the MOE values can help risk managers in defining the possible actions required to keep the acrylamide exposure as low as possible. Several methodologies are used to estimate the exposure to acrylamide, many of which rely on data from food consumption surveys and the databases of acrylamide content in different foods [9,10,11]. It is important to note that different countries, regions, or population groups may have different dietary habits and the levels of acrylamide in food may vary depending on the raw materials and culinary techniques used. However, these factors are often not taken into consideration when estimating the exposure to acrylamide. This fact highlights the need for more comprehensive and detailed assessments of the dietary exposure to this contaminant.

To better approximate the actual exposure to acrylamide, EFSA recommends the use of duplicate diet studies. These studies can improve the accuracy of the acrylamide-level assessments by providing more detailed information about the levels of the contaminant in foods, particularly those prepared at home or in public services or collectivities [8]. In this context, the aim of this research was to assess, for the first time in Spain, the acrylamide dietary exposure in university canteens with the duplicate diet method, estimating the associated risk of acrylamide exposure based on the food consumption habits of university students.

## 2. Materials and Methods

### 2.1. Chemicals and Reagents

Acrylamide standard (99%), potassium hexacyanoferrate (II) trihydrate (98%, Carrez-I), and zinc acetate dihydrate (>99%, Carrez-II) were purchased from Sigma (St. Louis, MO, USA). Formic acid (98%), methanol (99.5%), and hexane were obtained from Panreac (Barcelona, Spain). Acrylamide labelled by ^13^C_3_ (99%) was acquired from Cambridge Isotope Laboratories (Andover, MA, USA). To prepare all solutions, deionised water from a Milli-Q Integral 5 water purification system (Millipore, Billerica, MA, USA) was used. Cellulose syringe filter units (0.22 and 0.45 µm) were acquired from Análisis Vínicos (Tomelloso, Ciudad Real, Spain) and Oasis-HLB cartridges (30 mg, 1 mL) were obtained from Waters (Milford, MA, USA). All chemicals, solvents, and reagents used were of analytical grade.

### 2.2. Study Design and Food Samples

For the study, two university canteens located in Madrid (Spain) were selected (canteen 1 and canteen 2). Each canteen was associated with different universities and operated by separate companies, involving the different handling and preparation of food. The research was conducted between February and March 2020. Both canteens participated in a two-consecutive-week trial during which the foods and meals served for lunch between Monday and Friday were collected in accordance with the duplicate diet method [8] (Table 1). Both lunch menus were composed of a first course (first dish), a second course with a side dish (second dish), a dessert, and bread. When the canteen offered salad or French fries/baked rustic fries as options of side dishes, both foods were collected to address two exposure scenarios associated with the choice of consumption (the lowest exposure when salad was selected and the highest exposure when processed potatoes were selected). This allowed the researchers to assess the potential impact of different food choices by university students on acrylamide exposure. A total of 117 different foods/meals were collected. After collection, the food samples were coded, photographed, and processed in the laboratory. Each meal was assigned FoodEx 2 classifications according to EFSA [8,12], which is a standardised food classification system used to facilitate data collection and analysis. The FoodEx 2 system categorises foods based on their composition and culinary preparation, allowing for a more accurate assessment of acrylamide exposure. To prepare the food samples for analysis, the edible portion of each meal was separated, weighed, and homogenised using a hand blender (Taurus, vital CM, Spain). Each food/meal was homogenised separately and after that, the aliquots of each sample were stored at −20 °C until further analysis.

### 2.3. Acrylamide Determination by LC-ESI-MS/MS

The acrylamide content of foods and meals was determined according to the procedure described by González-Mulero et al. [13]. Two grams of each ground sample was weighted and mixed with 37.6 mL of Milli-Q water, 4 mL of hexane to remove the fat content, and 400 µL of a 5 µg/mL (^13^C_3_)-acrylamide methanolic solution as an internal standard. The mixture was homogenised and then treated with 1 mL of both Carrez I and Carrez II solutions and centrifuged. After the hexane removal, the samples were filtered through a 0.22 µm filter and cleaned using Oasis-HLB cartridges. Sample extracts and calibration standards were analysed on an Agilent 1200 liquid cromatograph coupled to an Agilent Triple Quadrupole MS detector (Agilent Technologies, Palo Alto, CA, USA). The precision, repeatability, and reproducibility of the analytical method were assessed by analysing different samples on the same day (precision), by different operators (repeatability), and on different days (reproducibility). The samples spiked with acrylamide showed a recovery rate ranging from 94% to 103%, with all determinations having relative standard deviations (RSDs) below 10%. To determine the limit of detection (LOD) and limit of quantification (LOQ) for the analytical method, lower concentrations of standards were injected, and their signal-to-noise ratios were calculated. The concentration that yielded a signal-to-noise ratio of 3 was assigned as the LOD (4.5 µg/kg), while the concentration that yielded a signal-to-noise ratio of 10 was assigned as the LOQ (15 µg/kg). The samples were analysed in duplicate and the results were reported in µg/kg.

To examine the accuracy of the findings, proficiency tests were conducted through the Food Analysis Performance Assessment Scheme (FAPAS) program. FAPAS^®^ Quality Control Test Materials are real food matrices used as quality control materials by laboratories participating in a proficiency test. The most recent results for the food matrices provided by FAPAS were for crispbread (test 30118, January 2022), coffee (test 30117, December 2021), and potato crisps (test 30120, April 2022). These tests yielded z-scores of −0.1, 0.1, and 0.4, respectively.

### 2.4. Estimation of Acrylamide Exposure

To determine the acrylamide exposure of university students (µg/kg bw/day) from consuming food and meals provided by lunches at university canteens, a deterministic approach was used. The acrylamide contribution of each meal for each individual was calculated by combining the acrylamide content of the meal with the weight of the edible portion of the serving. For samples with an acrylamide concentration below the LOQ (<15 µg/kg), their contribution to acrylamide intake was disregarded to avoid overestimating the acrylamide exposure, therefore, the lower bound scenario was estimated. The dietary acrylamide exposure of university students was calculated by adding up the acrylamide contribution of all the foods/meals consumed during a single day at lunch and then divided by their body weight (bw) (kg) (Equation (1)). An estimate was made of the average daily exposure over the 10-day study period.

(1) $$\mathrm{Estimated}\;\mathrm{dietary}\;\mathrm{exposure}=\Sigma \frac{\mathrm{acrylamide}\;\mathrm{content}\;\mathrm{of}\;\mathrm{food}\;\left(\frac{µg}{kg}\right)\;\times \;\mathrm{Food}\;\mathrm{consumption}\left(\frac{\mathrm{kg}}{\mathrm{day}}\right)}{\mathrm{Body}\;\mathrm{weight}}$$

The dietary exposure was estimated separately for males and females, using an average body weight data of 72 kg for males and 60 kg for females, as reported for a Spanish population aged 19–24 years [14]. The results were expressed as µg/kg bw per day.

To assess the risk of acrylamide exposure, a margin of exposure (MOE) was calculated for both neurotoxic and neoplastic effects, using the following formula:

(2) $$\mathrm{MOE}=\frac{BMDL10\left(µg/\mathrm{kg}\;\mathrm{per}\;\mathrm{day}\right)}{\mathrm{Estimated}\;\mathrm{dietary}\;\mathrm{exposure}\;(µg/\mathrm{kg}\;\mathrm{per}\;\mathrm{day})}$$

To assess the risk of neurotoxic effects, a benchmark dose lower confidence limit (BMDL10) of 430 µg/kg bw per day, previously calculated in rats [8], was used. MOEs below 125 may indicate a potential human health concern for neurotoxic effects. For carcinogenic effects, a BMDL10 of 170 µg/kg bw per day, previously calculated in male mice for Harderian gland tumours, was used. In this case, an MOE below 10,000 may indicate a potential risk [8].

### 2.5. Statistical Analysis

All statistical analyses were conducted using Statgraphics Centurion XVI.I software. The data were presented as means and standard deviations (SD). Student’s *t*-test was used to identify the overall significance of the differences. Levene’s test was used to check for variance homogeneity. All statistical analyses were carried out assuming a significance level of *p* < 0.05.

## 3. Results and Discussion

### 3.1. Acrylamide Content in Foods and Meals Provided by the University Canteens

Table 2 provides a summary of the acrylamide content in foods and meals served in the lunch at university canteens. Only 31% of the total 117 foods/meals analysed (36 samples) had acrylamide concentrations above the LOQ (15 µg/kg). The distribution according to the course type is as follows: 5 (13.9%) were first courses, 3 (8.3%) were second courses, 17 (47.2%) were side dishes, 10 (27.8%) were breads, and 1 (2.8%) was a dessert.

Considering only foods/meals with quantifiable acrylamide levels (Table 2), all the samples that were part of the first course, second course, and desserts showed low acrylamide concentrations, ranging from 15 µg/kg (chocolate custard) to 63 µg/kg (Spanish omelette with fried green pepper). The first courses consisted of stews or creams where the presence of acrylamide would not be expected. However, the preparation of these meals involves frying or sautéing certain vegetables, such as peppers, onions, or carrots, which can undergo the Maillard reaction during the cooking process. These vegetables contain sugars and asparagine, which promote the formation of the contaminant when exposed to heat [15,16]. Additionally, acrylamide may be introduced into the meals through ingredients commonly added to these dishes, such as spices or seasonings. Red paprika, turmeric, or black pepper, for example, have been reported to contain significant amounts of the contaminant [17,18]. Although in small quantities comparable to those observed in this study, the presence of acrylamide in soups, purees, and legume stews has been described by other researchers in previous studies [19,20]. As for the second courses, the acrylamide content in the Spanish omelette with green peppers could be attributed to both the fried potatoes included in the omelette [13] and the fried green peppers [21]. In the case of meatballs, it was likely linked to the contaminant content in the tomato sauce [22], although the contribution of other ingredients included in the meal, such as fried vegetables, cannot be ruled out as a potential source of acrylamide. Regarding desserts, only the chocolate custard showed detectable levels of this compound, which may be due to the use of chocolate as an ingredient in the recipe [23].

Low concentrations of acrylamide were also observed in the bread samples, with detectable levels only found in the breads from canteen 1 (mean value of 15 µg/kg). Results were significantly lower than the benchmark levels established by the European Regulation for wheat-based bread, which is set at 50 µg/kg [24]

The processed potatoes, including the French fries and baked rustic fries served as side dishes, showed the highest concentrations of acrylamide. Canteen 1 served French fries on the 10 days of the study, with acrylamide levels ranging from 16 to 1304 µg/kg. Meanwhile, canteen 2 offered potatoes as a side dish on 8 of the 10 study days, including French fries for 3 days and rustic potatoes for 5 days. The acrylamide levels varied from non-detectable (in one sample) to 67 µg/kg for French fries and between 93 and 351 µg/kg for rustic potatoes. Only the French fries sample from Monday of the first week in canteen 1 exceeded the reference value established by the European regulation for acrylamide in this food (500 µg/kg) [24]. Although French fries are typically linked to a higher acrylamide content than baked potatoes [25], the levels observed in this study were comparable between the two or even lower in French fries (average content: 185 µg/kg for French fries, considering levels from two canteens and 242 µg/kg for baked rustic fries). Previous studies about acrylamide in French fries prepared at Spanish school canteens and food service establishments reported higher concentrations (mean values: 329 and 303 µg/kg, ranges from <20 to 4000 and from <20 to 1068 µg/kg, respectively) [26,27].

To determine the real contribution of each food/dish to acrylamide exposure, the weight of the edible portion was considered (Table 2). The processed potatoes were the greatest contributors to acrylamide exposure, with the highest values found in baked potatoes (32.0 µg/serving), followed by French fries (27.2 and 4.2 µg/serving in canteen 1 and 2, respectively). The main courses contributed in a range between 2.6 and 22.6 µg/serving, while the lowest contribution was associated with the desserts and bread due to both lower levels of the contaminant and smaller portion sizes.

### 3.2. Acrylamide Intake Estimation in University Canteens under Different Scenarios

Table 3 displays the acrylamide intake levels calculated for each day of the week over the course of the two-week trial. The average values for both a one-week and a ten-day period of data collection were also calculated for each university canteen. Two scenarios were assessed, considering the selection of side dishes when two options are offered on the menu: the lowest exposure scenario, which assumes the selection of salad, and the highest exposure scenario, which assumes the selection of French fries/baked rustic fries. If the student chose to eat salad as long as it was included in the menu, the estimated acrylamide intake would range from 1.3 to 26.4 µg/day in canteen 1 and from negligible to 43.0 µg/day in canteen 2. On the contrary, if the student chose to eat processed potatoes, the acrylamide exposure would increase in canteen 1, ranging from 2.3 to 173.0 µg/day, not being affected in canteen 1 compared to the lowest exposure scenario. The mean acrylamide intake was not significantly different between the two canteens when comparing the lowest (*p* = 0.7751) or the highest exposure (*p* = 0.4702). Taking into account the two canteens together, the average daily intake of acrylamide could range from 6.5 to 26.4 µg, with significant differences (*p* = 0.0311), depending on whether the student chose salad or processed potatoes as side dishes.

**Figure 1:**
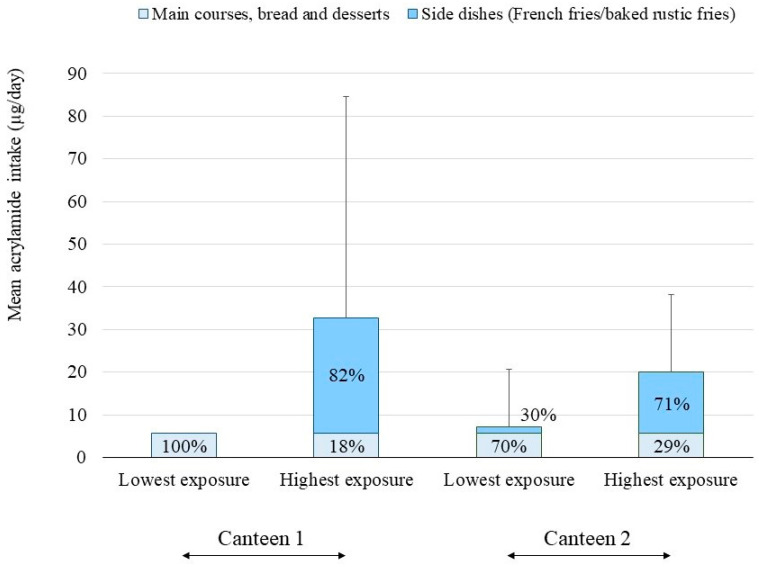
Mean daily acrylamide intake (µg) through the consumption of the menus provided by the university canteens and the contribution of side dishes and the rest of the foods/meals to the total daily intake.

In canteen 1, acrylamide was consumed daily due to the presence of the contaminant in the bread, which was included daily on the menu (Table 3). The intake increased when the first course also contained acrylamide and reached even higher levels when acrylamide was present in both the first and second courses (on Tuesday of the first week). The highest exposure in canteen 1 was associated with the consumption of French fries as a side dish, to such an extent that 82% of the daily consumption could be from this food if selected from the menu (Figure 1). The daily acrylamide intake could reach values of up to 173.0 µg/day in this scenario (Table 3). On the other hand, in canteen 2, five of the menus included in the study did not contain acrylamide levels. In this case, the maximum levels were reached when processed potatoes were included in the menu as the only alternative (Friday of the first week) or as the side dish chosen voluntarily (in the highest exposure scenario). The daily acrylamide intake could reach values of up to 43.0 µg (Table 3), assuming around 71% of the daily intake of the contaminant (Figure 1).

Although in recent years there has been an attempt to promote healthy eating habits among the general population, the consumption of fried, pre-cooked, or fast foods is still high, particularly among the younger population. Studies conducted on university students have revealed that the diet of the university population is of low quality, with a moderate-to-low adherence to the Mediterranean diet, where more than 80% of the population needs changes towards a healthier dietary pattern [28]. These habits include, among other factors, a high tendency to consume snacks and prepared meals even three or more times a week, including French fries [29]. A study carried out in a Spanish university canteen revealed a high tendency among young people to choose French fries as a side dish, with significant relationships found between the student’s gender and dish choice. Men ate more French fries than women (78% vs. 56.5%) and only 8.9% of them refused to have a side dish vs. the 35% of women [2]. A similar trend was observed among university students in Belgium, aged between 18 and 35 years. It was found that men ate French fries more frequently, usually a couple of times a month. However, the majority of women consumed French fries only occasionally or not at all likely due to a greater concern for a healthy diet and weight maintenance [30].

Our findings suggest that the regular consumption of French fries could increase the daily intake of acrylamide even by up to 92.5% (i.e., Monday, canteen 1, week 1). Therefore, this habit should be considered when evaluating the risks associated with the exposure to this contaminant. Furthermore, given the significant impact of frequent consumption of French fries in canteens on acrylamide intake, it is important to recognise the responsibility of caterers and canteen kitchens in mitigating acrylamide exposure by reducing the amount of this contaminant in their prepared products, particularly French fries, as previously suggested by Mestdagh et al. [30].

Researchers from Poland conducted a survey among a group of individuals between the ages of 16 and 35 to assess their knowledge regarding the presence of contaminants in the diet. The results showed that only 20% of the respondents take into consideration the potential content of harmful substances when choosing food products. With respect to acrylamide, only 7% of those surveyed reported being aware that this compound forms in food during its processing, with most having obtained this information from the Internet [31]. It is therefore necessary to not only promote the consumption of balanced diets among young people, but also to provide adequate training in food safety to encourage the selection of safe food options for consumers.

### 3.3. Evaluation of the Potential Risk Related to the Exposure to Acrylamide at the University Canteens

This study evaluated the mean dietary exposure to acrylamide in university students attending university canteens, separately for males and females and considering the scenarios of greater and lesser exposure to the contaminant (Table 4). The results showed that, taking into account the influence of body weight, women had a slightly higher acrylamide exposure compared to men, without significant differences (*p* < 0.05). When comparing the two canteens, as a consequence of the acrylamide levels found in the menus, the lowest exposure scenarios were similar in both food service establishments (range: 0.07–0.12 µg/kg bw per day). However, in the highest exposure scenarios, exposure to the contaminant was 40% lower in the second canteen (0.26 vs. 0.43 µg/kg bw per day in men and 0.33 vs. 0.54 µg/kg bw per day in women). Due to the high variability in the results, significant differences among the values could not be identified (*p* < 0.05).

Based on the overall data collected from both canteens (Table 4), the average exposure to acrylamide would be 0.08 and 0.11 µg/kg bw per day for men and women, respectively, when salad was selected as a side dish whenever it was offered on the menu (lowest exposure scenario). In contrast, exposure would change to 0.34 and 0.44 µg/kg bw per day for men and women, respectively, when the selected side dish was French fries/baked rustic fries (highest exposure scenario). The results were close to those described by Mestdagh et al. [30] when evaluating the dietary intake of acrylamide in the university canteen lunches of a Belgian university (mean acrylamide exposure: 0.537 µg/kg bw per day). It should be noted that this study only estimated the exposure to acrylamide from canteen lunches and did not take into account other sources of exposure from the rest of the diet throughout the day. Comparable findings regarding the dietary exposure to acrylamide among university students have also been reported in Ningxia (China), despite evaluating the complete daily diet of the students. The study found that the average exposure to the contaminant was 0.515 µg/kg bw per day (0.483 and 0.530 µg/kg bw per day for males and females, respectively), with potato products contributing 23.87% to the total exposure [32]. When three different exposure scenarios were assessed (low, middle, and high), our data were close to the middle scenario in the case of the highest exposure situation of canteen 1. Nevertheless, the highest exposure scenario in canteen 2 was closer to the low-exposure group considered by these researchers. They performed the estimation evaluating the total diet through a food frequency questionnaire and a database of acrylamide content in which food samples were purchased from different markets and then prepared according to several cooking methods [33]. Higher levels of acrylamide exposure have been reported in university students from Lima (Peru). In this case, the estimation was again made by applying a database of acrylamide content and a food frequency questionnaire evaluating the complete diet, reporting the exposure of 1.37 and 1.41 µg/kg bw per day in male and female students, respectively [34]. Most studies found in the literature use the methodology described above to assess the exposure to acrylamide through the diet. These studies usually rely on dietary surveys (such as 24 h recall, food dietary records, or food frequency questionnaires) to evaluate the food consumption and databases or the random sampling of foods to determine their acrylamide content [9,10,11]. However, these estimates may not take into account the significant variability that can exist in the acrylamide levels in processed foods, depending on factors such as the raw materials used or the specific culinary techniques employed [13] and the amount of food consumed by each individual, among others. To address these limitations, duplicate diet studies offer a more realistic estimate of the exposure and the associated risk by evaluating the real content of the contaminant and the precise amount of food consumed by a specific group [8]. The differences in the applied methodologies make it difficult to compare the results of the studies found in the literature. In that sense, it has been reported that the validity of the acrylamide intake estimation using a dietary record method could be reasonably high when compared to the analytical value of the duplicate diet method, with further improvement being required for estimating the acrylamide intake using food frequency questionnaires [35].

Acrylamide and its metabolite glycidamide are genotoxic and carcinogenic compounds due to their ability to potentially damage DNA and lead to cancer. The risk assessment for genotoxic compounds, for which no tolerable daily intake has been recommended, typically uses the margin of exposure (MOE) approach. An MOE above 10,000 is generally considered to indicate a low health concern for a compound with genotoxic and carcinogenic properties. In the same way, an MOE above 125 is generally considered to indicate the low health concern for a compound with neurotoxic properties [8]. In the present study, the MOE values suggest significant food health concerns for university students. The MOEs calculated for neurotoxic and neoplastic effects in university students are presented in Table 4. The MOE values for neurotoxic effects ranged from 982 to 5136, which were all above the reference value of 125, indicating a low health concern for these effects. However, the MOE values for neoplastic effects ranged from 388 to 2031, which were all well below the reference value of 10,000, indicating a high health concern for these effects with a higher risk in women than in men. Again, it is important to note that exposure was only estimated from lunch consumption, and thus, the total exposure to acrylamide is underestimated. This would further aggravate the concern regarding the high levels of exposure to acrylamide among university students. A similar trend was reported in university students from China, with MOE values exhibiting figures under 10,000 and even in some cases were below 200 but, in this case, assessing the total diet of the students [32]. According to the results, the risk of acrylamide exposure can increase by more than four times based on the food choices made by students. Therefore, it is crucial to promote healthy dietary habits during this stage of life when permanent patterns of nutritional behaviour are being constructed and implement suitable mitigation measures while cooking in public canteens.

## 4. Conclusions

This investigation focused on the evaluation of dietary exposure to acrylamide in university canteens, estimating the associated risk based on the food consumption habits of university students by establishing a maximum and a minimum scenario of exposure to the contaminant. As recommended by EFSA, a duplicate diet study was designed to better approximate to the actual exposure to acrylamide considering the real portions and analysing the acrylamide concentrations in all the collected foods/meals provided in the lunches of the university canteens. Most of the analysed food preparations either did not contain detectable levels of acrylamide or had low levels. However, the processed potatoes were found to be the primary source of the contaminant, which could result in an increased daily acrylamide exposure if they are selected as a side dish when offered on the menu. Due to their lower body weight, women had higher acrylamide exposure compared to men. The calculation of the MOE values for neoplastic effects indicated a high health concern, with a higher risk in women than men, even considering only the central meal of the day. Moreover, the risk of acrylamide exposure can increase by more than four times depending on the side dish selected by students. Our research establishes the need to promote healthy dietary habits among young people, but also to provide proper training in food safety targeting both consumers and food handlers to encourage the selection of safer food options in terms of food processing contaminants.

## Figures and Tables

**Table 1:** Description of the menus collected from the university canteens.

		Monday	Tuesday	Wednesday	Thursday	Friday
**University Canteen 1**	**Lunch Week 1**	Lentils stew Cod fish in tomato sauce French fries/mix salad Pudding Bread	Garlic soup with bread and poached egg Battered squid rings French fries/mix salad Orange Bread	Spaghetti with garlic Chicken stew French fries/mix salad Flan Bread	Rice stew with vegetables Sauteed green asparagus with scrambled eggs French fries/mix salad Apple Bread	Bean stew Grilled swordfish French fries/mix salad Yoghurt Bread
	**Lunch Week 2**	Braised broccoli with garlic Fried eggs French fries/mix salad Rice pudding Bread	Canned white asparagus with salad Fried blue whiting French fries/mix salad Chocolate and cream cup Bread	Spaghetti carbonara Grilled chicken French fries/mix salad Apple Bread	Potatoes with cod stew Roasted pork loin French fries/mix salad Pudding Bread	Vegetables cream Meat lasagne French fries/mix salad Yoghurt Bread
**University Canteen 2**	**Lunch Week 1**	Mixed paella Pork loin in tomato sauce Baked rustic fries/mix salad Strawberry jelly Bread	Pumpkin cream Baked pomfret French fries/mix salad Coconut mousse Bread	Sautéed green beans Spanish omelette with fried green pepper Mix salad Custard with biscuit Bread	Fried spring rolls Chicken stew Baked rustic fries/mix salad Banana Bread	Mixed salad Red wine-braised sausages Baked rustic fries Orange Bread
	**Lunch Week 2**	Vegetable and garlic cream Meatballs in tomato sauce Baked rustic fries/mix salad Yoghurt Bread	Sauteed vegetables Cod fish in tomato sauce Boiled white rice Rice pudding Bread	Pasta with tomato sauce Baked redfish with sautéed vegetables French fries/mix salad Chocolate custard Bread	Pasta salad Pork loin in tomato stew French fries/mix salad Strawberry jelly Bread	Vegetables cream Grilled mackerel Baked rustic fries/mix salad Melon Bread

**Table 2:** Acrylamide content in foods/meals compounding lunches of university canteens.

Meal	Foods	n ^a^	FoodEx 2 Code	Acrylamide Content (µg/kg)	Edible Serving (g)	Acrylamide Contribution (µg/Serving)
**Canteen 1**						
**First course**	Lentils stew	1	A00QD#F28.A07GM	31	378	11.6
	Garlic soup with bread and poached egg	1	A041Z#F04.A0DRD$F04.A00GZ	52	432	22.6
	Bean stew	1	A03VR	22	334	7.4
**Second course**	Battered squid rings	1	A02JJ#F28.A07HL	17	149	2.6
**Side dish**	French fries	10	A0BYV	221 ± 444	120 ± 32	27.2 ± 54.4
	Bread	10		15 ± 0.9	90 ± 8	1.3 ± 0.3
**Canteen 2**						
**First course**	Vegetable and garlic cream	1	A03XY#F04.A00GZ	18	241	4.3
	Vegetable cream	1	A03XY	18	252	4.6
**Second course**	Spanish omelette with fried green pepper	1	A03YN#F04.A00ZT#F04.A019A#F28.A07GR	63	225	14.2
	Meatballs in tomato sauce	1	A03XG#F04.A044C	23	172	4.0
**Side dish**	French fries	3	A0BYV	41 ± 37	112 ± 20	4.2 ± 3.4
	Baked rustic fries	5	A011R	242 ± 120	134 ± 13	32.0 ± 14.6
**Dessert**	Chocolate custard	1	A02PX#F04.A034L	15	115	1.8

Only dishes with an acrylamide concentration higher than LOQ (15 µg/kg) are detailed (36 from a total of 117 foods/meals analysed). ^a^ n refers to the times that foods/meals were collected during the study period. Data are expressed as mean ± SD.

**Table 3:** Acrylamide intake through the consumption of the menus provided by the university canteens.

Acrylamide Intake (µg)						
LOWEST EXPOSURE						
	Monday	Tuesday	Wednesday	Thursday	Friday	Mean
Canteen 1						
Week 1	13.0 ± 0.2	26.4 ± 0.7	1.4 ± 0.1	1.3 ± 0.1	8.6 ± 0.9	10.1 ± 10.4
Week 2	1.3 ± 0.1	1.4 ± 0.1	1.3 ± 0.1	1.4 ± 0.1	1.3 ± 0.1	1.3 ± 0.1
Daily mean (10 days)						5.7 ± 8.3 a
Canteen 2						
Week 1	nd	nd	14.2 ± 1.0	nd	43.0 ± 0.7	11.5 ± 18.7
Week 2	8.3 ± 0.3	nd	1.8 ± 0.1	nd	4.6 ± 0.5	2.9 ± 3.5
Daily mean (10 days)						7.2 ± 13.5 a
Both canteens						6.5 ± 1.1 A
HIGHEST EXPOSURE						
	Monday	Tuesday	Wednesday	Thursday	Friday	Mean
Canteen 1						
Week 1	173.0 ± 2.6	52.9 ± 1.4	8.0 ± 0.1	27.8 ± 0.7	21.6 ± 1.9	56.7 ± 67.1
Week 2	28.6 ± 0.5	5.2 ± 0.1	3.5 ± 0.1	5.3 ± 0.3	2.3 ± 0.1	9.0 ± 11.0
Daily mean (10 days)						32.8 ± 51.8 a
Canteen 2						
Week 1	42.8 ± 0.2	4.5 ± 0.3	14.2 ± 1.0	12.0 ± 0.4	43.0 ± 0.7	23.3 ± 18.2
Week 2	38.4 ± 0.9	nd	8.3 ± 0.3	nd	37.0 ± 1.3	16.7 ± 19.4
Daily mean (10 days)						20.0 ± 18.1 a
Both canteens						26.4 ± 9.1 B

nd: non-detected. Data are expressed as the total intake each day of the study and daily mean during the whole 10-day period ± standard deviation considering only lunches. The lowest exposure involves the selection of salad as side dishes when offered in the menu. The highest exposure involves the selection of French fries/baked rustic fries as side dishes when offered in the menu. Same lowercase letters in the values for the daily mean (10 days) involve no significant differences between schools within each exposure scenario. Different uppercase letters in the values for both canteens involve significant differences between the lowest exposure and highest exposure.

**Table 4:** Estimation of the risk associated with dietary exposure to acrylamide in lunches of university canteens.

	Men		Women	
	Lowest Exposure	Highest Exposure	Lowest Exposure	Highest Exposure
Faculty canteen 1				
Mean exposure ± SD (µg/kg bw/day)	0.07 ± 0.11 aA	0.43 ± 0.67 aA	0.10 ± 0.14 aA	0.54 ± 0.86 aA
MOE for neurotoxic effects	5791	1011	4525	790
MOE for neoplastic effects	2289	400	1789	312
Faculty canteen 2				
Mean exposure ± SD (µg/kg bw/day)	0.09 ± 0.17 aA	0.26 ± 0.23 aA	0.12 ± 0.22 aA	0.33 ± 0.30 aA
MOE for neurotoxic effects	4615	1658	3606	1296
MOE for neoplastic effects	1824	656	1426	512
Both university canteens				
Mean exposure ± SD (µg/kg bw/day)	0.08 ± 0.14 aA	0.34 ± 0.50 aA	0.11 ± 0.18 aA	0.44 ± 0.64 aA
MOE for neurotoxic effects	5136	1257	4014	982
MOE for neoplastic effects	2031	497	1587	388

SD: standard deviation. Same lowercase letters involve no significant differences between lowest and highest exposure for a same sex in each canteen. Same uppercase letters involve no significant differences between sex for each exposure scenario.

## Data Availability

The authors do not have permission to share data.
